# Implementing a digital mental health intervention for individuals with psychosis - a multi-country qualitative study

**DOI:** 10.1186/s12888-021-03466-x

**Published:** 2021-09-25

**Authors:** Tamara Pemovska, Aliriza Arënliu, Jon Konjufca, Fitim Uka, Jennifer Hunter, Stojan Bajraktarov, Lidija Injac Stevović, Stefan Jerotić, Alma Džubur Kulenović, Antoni Novotni, Ljubisa Novotni, Tamara Radojičić, Selman Repišti, Emina Ribić, Ivan Ristić, Eldina Smajić Mešević, Mirjana Zebić, Nikolina Jovanović

**Affiliations:** 1 Unit for Social and Community Psychiatry, Queen Mary University of London, London, E13 8SP UK; 2 Department of Psychology, University of Pristina, 10000 Pristina, Kosovo; 3 School of Health Sciences, City University of London, London, EC1R 1UB UK; 4 University Clinic of Psychiatry, Ss. Cyril and Methodius University in Skopje, Skopje, 1000 North Macedonia; 5 Clinical Centre, Psychiatric Clinic, University of Montenegro, Podgorica, Montenegro; 6 Faculty of Medicine University of Belgrade & Clinic for Psychiatry, University Clinical Centre of Serbia, Belgrade, Serbia; 7 Department of Psychiatry, Clinical Centre of the University of Sarajevo, 71000 Sarajevo, Bosnia and Herzegovina

**Keywords:** Implementation science: psychosis, Mental health care, Psychosocial intervention, Digital intervention, Southeast Europe, Low- and middle-income setting, Multi-country qualitative research

## Abstract

**Background:**

Implementation of psychosocial interventions in mental health services has the potential to improve the treatment of psychosis spectrum disorders (PSD) in low- and middle-income countries (LMICs) where care is predominantly focused on pharmacotherapy. The first step is to understand the views of key stakeholders. We conducted a multi-language qualitative study to explore the contextual barriers and facilitators to implementation of a cost-effective, digital psychosocial intervention, called DIALOG+, for treating PSD. DIALOG+ builds on existing clinician-patient relationships without requiring development of new services, making it well-fitting for healthcare systems with scarce resources.

**Methods:**

Thirty-two focus groups were conducted with 174 participants (patients, clinicians, policymakers and carers), who were familiarized with DIALOG+ through a presentation. The Southeast European LMICs included in this research were: Bosnia and Herzegovina, Kosovo, (Kosovo is referred throughout the text by United Nations resolution) North Macedonia, Montenegro and Serbia. Framework analysis was used to analyse the participants’ accounts.

**Results:**

Six major themes were identified. Three themes (Intervention characteristics; Carers’ involvement; Patient and organisational benefits) were interpreted as perceived implementation facilitators. The theme Attitudes and perceived preparedness of potential adopters comprised of subthemes that were interpreted as both perceived implementation facilitators and barriers. Two other themes (Frequency of intervention delivery; Suggested changes to the intervention) were more broadly related to the intervention’s implementation. Participants were exceedingly supportive of the implementation of a digital psychosocial intervention such as DIALOG+. Attractive intervention characteristics, efficient use of scarce resources for its implementation and potential to improve mental health services were seen as the main implementation facilitators. The major implementation barrier identified was psychiatrists’ time constrains.

**Conclusions:**

This study provided important insights regarding implementation of digital psychosocial interventions for people with PSD in low-resource settings by including perspectives from four stakeholder groups in five LMICs in Southeast Europe – a population and region rarely explored in the literature. The perceived limited availability of psychiatrists could be potentially resolved by increased inclusion of other mental health professionals in service delivery for PSD. These findings will be used to inform the implementation strategy of DIALOG+ across the participating countries. The study also offers insights into multi-country qualitative research.

**Supplementary Information:**

The online version contains supplementary material available at 10.1186/s12888-021-03466-x.

## Background

Effective treatment and management of psychotic spectrum disorders (PSD) is multi-faceted and expensive [1, 2]. PSD frequently cause severe disability, more often than other mental health disorders, resulting in significant health, social and economic burden [1].

In many countries such as the United Kingdom (UK), evidence-based treatment of PSD involves pharmacotherapy, family education and psychosocial interventions [3]. However, care for PSD in low- and middle-income countries (LMICs) in Southeast Europe (SEE) is characterized by meetings between clinicians and patients dominated by pharmacotherapy while psychosocial aspects tend to be overlooked, reportedly because of the lack of qualified staff and sufficient funding of mental health services [4, 5]. This facilitates additional inequalities of an already highly vulnerable population.

Cost-effective and easily deliverable digital psychosocial interventions embedded on a device, such as an application on a tablet [6], offer an opportunity to advance mental health care to people affected by PSD in LMICs and to alleviate the burden of psychosis on patients and their families.

One such intervention is DIALOG+, which is based on patient-centred communication, quality of life research and solution-focused therapy and designed to make routine mental health clinical meetings more therapeutically effective. The intervention is available as an app and uses a tablet computer. Each session begins with the patient rating their satisfaction with eight life areas and three treatment areas on a DIALOG scale of 1 (totally dissatisfied) to 7 (totally satisfied). The ratings are displayed on the tablet screen, allowing for comparisons with ratings from previous sessions. Each rating is reviewed through positive reinforcement and particular areas of concern are addressed in more detail in a four-step, solution-focused approach which aims to identify and utilize the patient’s existing resources [7]. Additional file 1 contains visual representations of DIALOG+.DIALOG+ builds on the existing clinician-patient relationships, which do not require formation of new services or referrals to other clinicians, making it a good fit for healthcare systems with scarce resources. After 3 h of training, a range of clinicians have been shown to be able to use the intervention [8]. DIALOG+ has proved effective in decreasing clinical symptoms, increasing quality of life and reducing treatment costs of patients with psychosis in the UK [8]. A pilot study in Germany also showed significant amelioration of schizophrenia and depressive symptoms as well as quality of life [9]. A process evaluation of the intervention identified the following likely ‘active ingredients’: 1) solution-focused structure; 2) patient empowerment; 3) mobilizing social resources; and 4) self-reflexion and therapeutic expression [10]. DIALOG+ has been implemented as a care planning tool and patient-reported outcome measure in health institutions in England and Wales [11–13]. It is also being researched in implementation and effectiveness studies across four continents [9, 14, 15], emphasizing the flexible use of the intervention.

Translating evidence-based interventions from high-income countries (HICs) to LMICs requires an in-depth understanding of contextual barriers and facilitators to implementation [16], factors which should be considered before implementation of any novel intervention to ensure the greatest chance of a successful outcome [17, 18]. The current literature from HICs points that research about digital psychosocial interventions has mainly focused on individual or intervention level implementation barriers. Findings indicate that attitudes and beliefs about interventions are crucial for staff and service users, and that the complexity of interventions is an important barrier for implementation of digital psychosocial interventions [19]. Implementation barriers identified in LMICs are more on the supply side, such as low staff/patient ratio, and on services users’ side, such as low digital literacy and poor wireless connectivity limiting usage of some mental health apps [20].

Therefore, this study aims to identify the perceived implementation facilitators and barriers of an evidence-based, digital, psychosocial intervention for patients with psychosis in five LMICs in SEE. The findings could provide evidence about the provision of digital psychosocial treatment for PSD in low-resource mental health settings and facilitate successful uptake of such interventions.

## Methods

### Design

This is a multi-country, multi-language qualitative study of the perceived implementation feasibility of DIALOG+. It involved primary data collection using qualitative methods in the form of semi-structured focus groups to explore perceived implementation facilitators and barriers of DIALOG+ from the perspective of key stakeholders, patients with psychosis, carers, clinicians and policymakers, in five SEE countries (Bosnia and Herzegovina, Kosovo^1^, North Macedonia, Montenegro and Serbia). Additional file 2 contains SRQR reporting guidelines [21].

The study is part of the larger research project “Implementation of an effective and cost-effective intervention for patients with psychotic disorders in low and middle-income countries in Southeast Europe” - IMPULSE, which is funded by the European Commission [22].

### Data collection

#### Participants

The research included 174 participants who participated in 30 focus groups and two interviews. The participants were a mix of patients, carers, clinicians and policymakers (Table 1). There were a higher proportion of female participants (*n* = 108; 62%) and most participants were patients (*n* = 59; 34%) followed by clinicians (*n* = 47; 27%), caregivers (*n* = 40; 23%) and policymakers (*n* = 28; 16%).

**Table 1:** Number of focus groups (FGs) and study participants per country

		Clinicians	Policymakers	Patients	Carers	Total number (%)
**Bosnia and Herzegovina**	FGs	2	2	2	2	8
	Participants	10	5	10	10	35 (20%)
**Kosovo**	FGs	1	1	2	1	5
	Participants	8	6	16	8	38 (22%)
**North Macedonia**	FGs	2	1	2	1	6
	Participants	13	6	15	6	40 (23%)
**Montenegro**	FGs	2	1	2	2	7
	Participants	10	7	12	11	40 (23%)
**Serbia**	FGs	1	1 FGs 2 interviews	1	1	4 FGs 2 interviews
	Participants	6	4	6	5	21 (12%)
**TOTAL**	FGs	8	6 FGs 2 interviews	9	7	30 FGs 2 interviews
	Participants	47 (27%)	28 (16%)	59 (34%)	40 (23%)	174

Clinicians, patients and carers were purposively recruited from mental health services, service user organisations and mental health non-governmental organisations. Policymakers and service manages were purposively recruited from Ministries of Health, National Psychiatric Associations and mental health medical boards. Patients’ inclusion criteria were: 18 to 65 years of age; in psychiatric treatment for at least 3 months; clinical diagnosis of psychosis or related disorder (i.e., ICD-10 F20-29, F31); and capable of giving informed consent. The inclusion criteria for clinicians were: professional qualification working with mental health services and more than 6 months working experience in mental health. Patients’ carers were included if they were: at least 18 years of age; a carer for someone with mental health difficulties or providing support outside of clinical context; and capable of giving informed consent. The inclusion criteria for policymakers and services providers were managerial and/or policymaking experience in health care, local or national authorities dealing with mental health services and policies.

Thirty focus groups were conducted: eight with clinicians, six with policy makers, nine with patients and seven with carers. Two policymakers were interviewed because they were unable to attend the focus groups due to other commitments (Table 1). For the sample size, we were guided by published recommendations that 5 to 8 participants in focus groups is adequate for achieving saturation of data and allowing all participants to express their thoughts and ideas in focus group studies [23]. A comprehensive description of the setting and contextual factors where data were collected is included elsewhere [15].

#### Topic guides

The topic guides explored participants’ views on barriers, facilitators and perceived benefits of implementing DIALOG+. They were based on the same set of topics for patients/family members and clinicians/policymakers, but with probes specific for each group (file 3). The same topic guide was used during the interviews with a policymakers.

The topic guides were developed in English in an iterative process, and draft versions were circulated among all collaborating centres for feedback. The final versions of the topic guides were translated into the national languages.

#### Procedure

The focus groups lasted 59.4 min on average and used similar methodology in the following sequence: 1) introduction and explanation of the aim of the focus group; 2) standardized presentation about DIALOG+, lasting 10 min on average; 3) focus group discussion related to barriers and facilitators to implementation of DIALOG+.

Focus groups took place between July and November 2018, in private rooms at the collaborating SEE mental health centres by male and female facilitators, who were local medical doctors and/or psychologists. Additional training in facilitating focus groups prior to data collection was provided to ensure a standardized approach.

All focus groups were audiotaped; transcribed verbatim and de-identified. The transcripts were translated to English either by local researchers proficient in the English language or by professional translators.

### Ethical procedures

Research activities followed current legislation and regulations in the participating SEE centres and the guidelines provided in Declaration of Helsinki. Each SEE collaborating centre obtained ethical approval from their local ethical committee prior to study beginning: Ethics committee of the Clinical Centre of the University of Sarajevo, Bosnia and Herzegovina (Ref: 03-02-47500, date approved: 13/09/2018); Ethical Professional Committee, Hospital and University Clinical Service of Kosovo, University Clinical Centre of Kosovo (Ref: 904, date approved: 08/06/2018); Ethical Committee for Research with Humans, Medical Faculty at the University of Cyril and Methodius in Skopje, North Macedonia (Ref: 03-2237/12, date approved: 21/05/2018); Ethics Committee for the Clinical Centre of Montenegro, Montenegro (Ref: 03/01-11066/1, date approved: 19/07/2018); Ethical Committee of the University of Belgrade Faculty of Medicine (Ref: 2650/V1-3, date approved: 26/06/2018). The local researchers provided detailed study information to potential participants and obtained written informed consent for research participation before data collection.

### Data analysis

Prior to analysis all data revealing personal identities was removed. We analysed data using thematic framework analysis [24] suitable for the large breadth of our dataset, collected in five different languages. Our research question was classified within the “diagnostic” and “strategic” typologies defined by Ritchie and Spencer [24] because we were interested in identifying both perceived barriers and facilitators to DIALOG+ implementation (diagnostic) and how they could potentially be addressed (strategic) [24, 25].

Four researchers, with previous background in qualitative studies, conducted the data analysis in Kosovo and in the UK (AA, JK, TP and JH). First, we familiarized ourselves with all the transcripts and each researcher created a preliminary coding list in light of our research question to identify perceived facilitators and barriers as well as any other codes developed and interpreted from the data. The individual preliminary coding lists were combined in a single one created by consensus through face-to-face discussions. The coding list was distributed to the focus group facilitators to check for accuracy. Additional file 4 contains the coding list.

Next, we grouped the preliminary codes to develop broad framework categories based on both concerns related to our research question and other emerging ones from the familiarization stage. Upon agreement of the framework, two researchers coded focus groups from clinicians and policy makers, and two other researchers coded focus groups from patients and carers using ATLAS.ti qualitative analysis software. Researchers coded the same transcripts until there were no discrepancies between them.

After organizing the data according to the framework categories, they were summarized for each category in a chart form. This was followed by mapping and interpretation of the data to find patterns. This process was on going until no additional patterns were found.

Local Lived Experience Advisory Panels (LEAP) were formed in each country to involve patients and carers as experts by experience in the research. Feedback from LEAP members was collected during regular LEAP IMPULSE project meetings in all participating countries. The LEAP provided feedback on the findings from focus groups with patients and carers. LEAP members did not participate in the focus groups. Members were given material, containing a summary of the methods and titles of the themes identified thus far from focus groups with patients and carers, several days prior to the meeting. Local researchers collected feedback from the LEAP members on the materials provided, based on pre-specified questions (same for all countries), which was summarized in the current study.

## Results

The results present the major themes related to the research aim to identify perceived facilitators and barriers to DIALOG+ implementation by four key groups of stakeholders. Two developed themes (Frequency of intervention delivery; Suggested changes to the intervention) are more broadly associated with DIALOG+ implementation. Differences between participant groups are explored within the identified themes. Views from the interviewed persons were in line with views elicited in focus groups.

**Fig. 1:**
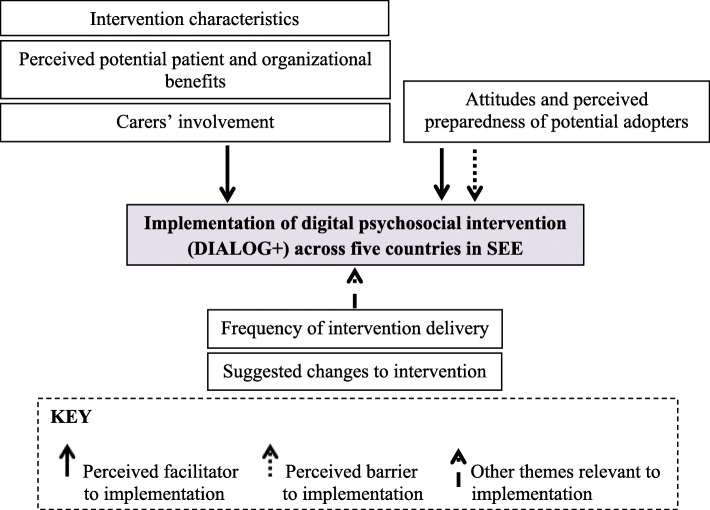
Major themes related to the implementation of DIALOG+ across participating countries in Southeast Europe (SEE). Adapted from Yang et al., 2015 [36]

Six major themes were developed: Intervention characteristics; Attitudes and perceived preparedness of potential adopters; Carers’ involvement; Perceived potential patient and organisational benefits; Frequency of intervention delivery; and Suggested changes to the intervention (Fig. 1).

Each major theme was developed from subthemes, summarized in Table 2.

**Table 2:** Summary of themes and subthemes

Theme	Subthemes
**Intervention characteristics**	Clarity and easiness of use
	Involving patients’ psychosocial needs
	Structuring routine clinician-patient meetings
	Ability to track patients’ progress
**Attitudes and perceived preparedness of potential adopters**	Implementation resources (staff, funding, time, space)
	Consistency with existing norms, policies and procedures
	Motivation to implement across potential adopters
	Potential adopters’ resistance to innovation
	Level of existent skills and relevant experience by potential adopters
	Perceived need for the intervention
	Institutional support
	Influence of poor mental health of patients
**Carers’ involvement**	Role of carers in intervention implementation
	Extent to carers involvement
**Perceived potential patient and organisational benefits**	Patient empowerment
	Improved therapeutic relationship
	Reinforcement of psychosocial care approach
	Standardization of care across services
	Reinforcing patient-centred care
	Improving structure and follow-up of care
**Frequency of intervention delivery**	Monthly delivery
	More frequent delivery than monthly
**Suggested changes to the intervention**	Changes to the DIALOG scale
	Changes to interpretation of life areas
	Changes to mode of intervention delivery

### Intervention characteristics

The intervention’s characteristics, likely the starting point of adopters’ engagement with the intervention, were largely interpreted as facilitators to the implementation across participant groups. Clinicians, patients and policymakers viewed DIALOG+ as clear and easy to use. Clinician45 from Serbia expressed “[DIALOG+] couldn’t be simpler”. Yet, a few patients expressed ambiguity when asked about their understanding of the DIALOG+ rating scale and its items. Previous familiarity with Likert scales and quality of life measures is likely to influence how easily patients can understand the rating scale that is part of the intervention.

Patient31: Yes, [DIALOG+ rating scale] is a classic Likert scale.

Patient30: [About the rating scale] when there are such gradations, one gets confused. I think there is no need for more than five [options]. It should be simpler.

Clinicians, patients and policymakers viewed DIALOG+ as adding structure to routine clinician-patient meetings while involving the psychosocial needs of the patient. Clinicians spoke about DIALOG+ creating a workflow for patients and clinicians that could lead to more comprehensive therapeutic approaches. Regularly using DIALOG+ would make it less likely for clinicians to omit topics that might be relevant to treatment.

Clinician9: I like that this intervention is well-structured, involving all the most important areas of one’s life and visual approach that doesn’t let us forget some domain.

Clinicians, patients and carers found the ability of the intervention to track patients and their treatment progress as particularly attractive.

Carer11: [With DIALOG+] there will be a follow up of [the patient’s] condition, which is the most important thing for the patient, number one issue (…) Simply, his condition is monitored, how is it today, is it getting worse or better, are there any improvements.

### Attitudes and perceived preparedness of potential adopters

Some patients, policymakers and carers expressed that the resources to implement DIALOG+ in their facilities are readily usable and available.

Patient19: The medical personnel are sufficient [to implement DIALOG+]. Policymaker3: [DIALOG+] is basically free. There are lots of things that I would like to implement here, but they are expensive (…) But this can be delivered by nurses as well.

However, when asked about resources that might be lacking, other participants expressed serious concerns about the limited resources available, particularly regarding time, staff, funding and space. Conflicting views regarding the existing resources to implement the intervention may reflect resource differences in the explored services.

Patient2: Medical staff of this clinic, they are great, but there just aren’t enough of them.

Carer39: I will give a specific example [of a usual clinical meeting] in my experience as both a caregiver and a patient. The doctor asks me – “Are things better? Are life’s problems worse than before?” but I try to make my conversations with the doctor as brief as possible. I can see there are so many patients waiting to be examined. I can see that patients come from all over the country with their bags, waiting to be examined. So with the doctor, I speak shortly and concretely. [DIALOG+] is something else; this is likely to last longer.

Policymaker26: In Serbia, it is practically impossible in the conditions of usual ambulatory controls, we do not have time for it, really.

Clinician34: You have to give tablet computers to every clinician and pay for tablets and software and everything else. If there is no money for it, how...

Clinicians, patients and policymakers viewed DIALOG+ as consistent with existing norms, policies and procedures in their services. However, participants from Serbia and North Macedonia questioned the suitability of DIALOG+ with the current funding policies that prioritize the quantity of patients examined. Furthermore, some policymakers expressed that implementing DIALOG+ might lead to “loss of individualized approach” to patients. This is likely motivated by the difference between the current, largely unstructured clinical meetings in mental health care compared to the structured procedure of using DIALOG+ supported by technology. It is interesting that the same characteristics of the digital intervention which are considered to dehumanize the intervention by some are also highly appreciated by others.

Policymaker25: DIALOG+ would fit well, I think, very well in the current practice, because, it is actually a modification of the practice that is already being done, but it is more effective and faster.

Policymaker2: I am just afraid of robotizing sessions after certain period of time.

Opposing views were expressed regarding the motivation across participants and peers to implement DIALOG+. Some participants expect that there will be clinicians’ resistance to innovation. Such views likely reflect the context of mental health services in the countries, where digital interventions are not a common practice.

Clinician9: I think that [using DIALOG+] is related to the [clinician’s] individual approach; surely some of our colleagues would be thrilled.

Policymaker4: As a member of a management board, I strongly believe that this can be implemented in our routine practice when we do all the legal work needed for something to become a routine intervention … certification, coding, and setting up the price. I am definitely in for implementing this.

Patient5: I could use it every time … I need to talk more, to tell what’s on my mind, but the doctor never has enough time.

Policymaker2: I think opinions would be divided. You cannot expect all [clinicians] to accept something; some people have resistance towards technology …

For most clinicians in the study it is vital that their service managers support them in the implementation of DIALOG+. Clinician14 stated “I think that we must first have the support of management”.

Additionally, all participant groups reported that skills and experience to implement DIALOG+ by key stakeholders are already existent. Some clinicians and patients asked for more information on how to use DIALOG+. Participants only received a short presentation describing the intervention, which is likely the motivation for such accounts. A few patients reported the need for training due to their lack of familiarity with smart phones/tablets.

Clinician3: We would have to get some instructions, this presentation was great, but we would need detailed training.

Policymaker8: No, [DIALOG+] won’t be different [in the skills required] (…) all of these [clinicians] are trained and they talk to patients – some for therapy, some for supportive psychotherapy – and each of them has his own way.

Patient3: I am good with computers, this is too easy.

Patient10: I don’t use a smart phone … so I would need [training].

An important implementation facilitator interpreted from the data is the participants’ perceived need for DIALOG+ implementation, particularly as it supports innovation in mental health care. Patients and carers reported that the current clinical meetings are too short to fully understand the patient and thus are limiting in the scope of conversation.

Patient45: [Clinicians] also pay little attention to the patient during the appointments, only 10–15 min or 5, irrelevant, but I think [DIALOG+] is already better.

Clinician9: I think that [DIALOG+] is very useful, that is exactly what we need.

Policymaker17: In the new National Strategy that is under preparation, we have included a mental health category. So this project comes at the right time so that we can think about which services we can develop. We are very thin in this field. It should start inter-sector cooperation at the local level. This project can help us a lot …

Carer26: I think it will succeed, [implementing DIALOG+] is a step forward in medicine, having in mind how patients are treated, all is done in the old fashioned way, and nothing has improved until now.

Participants expressed that poor mental health of patients can be a barrier to DIALOG+ implementation. Policymaker2 said that “[DIALOG+ areas are significant when patients reach remission, when they can handle real-life situations.” This result highlights that the intervention may not be suitable for all patients and some selection criteria may be necessary.

### Carers’ involvement in intervention

Participants expressed varying views regarding involvement of carers in DIALOG+ implementation, although all participant groups saw carers to have a facilitating role. Clinicians and carers proposed carers to be involved in the planning and execution of actions agreed from the sessions. Clinicians expressed that DIALOG+ could offer carers psycho-education. However, patients and clinicians also showed reluctance about the extent to carers’ involvement during sessions. The accounts suggest that the level of carers’ involvement with such an intervention requires adjustment to the patient’s needs.

Clinician10: We should just keep caregivers away from assessing areas instead of patients.

Policymaker3: I think caregivers should take part in this. Whether they take part on the sessions or afterwards … it depends on what the patient wants...

Patient8: I would like [family members to be involved] in that part where doctor gives tasks to family members and me. I wouldn’t like them there all the time.

Patient56: [Carers] could encourage and stimulate clients, and at the same time they could help clinicians be better informed about their clients’ problems – from a different point of view. I think carers could understand and explain certain issues more objectively than clients themselves.

### Perceived potential patient and organisational benefits

Clinicians, patients and carers reported patient empowerment and an improved therapeutic relationship as potential benefits of the intervention to the patient. Other potential benefits at an organisational level were also reported: DIALOG+ would help shift the mental health services away from a typical medical approach to care by reinforcing a psychosocial care approach (clinicians, patients, carers); care could become more patient-centred (patients and carers); the structure and follow-up of care could be improved (all participant groups), and DIALOG+ could offer an opportunity for care to be standardized across services (by policymakers). These subthemes reflect how DIALOG+ could add to the routine service delivery and the elicited views are likely related to what participants are missing from their current care.

Carer36: The clinician gets closer to the patient, and the patient increases his confidence in the clinician [with DIALOG+]. If this intervention goes on for a longer time, this can only deepen and expand the relationship.

Patient3: I like that we have freedom, that we can follow this app and that we have information from previous sessions. We see if we have made progress or not, we can see if the doctor’s therapy, advice, or drug were adequate.

Patient56: It would motivate me to work harder on myself and spend less time doing nothing but wandering around.

Policymaker2: [DIALOG+] would be good because we would standardize appointments in time, content, and approach.

Clinician3: It increases functionality in every aspect, what is important for the patient, engages patients in decision-making process, motivates them. That increases self-confidence and empowers the patient.

Clinician45: The mere fact that conversations with my patients would cover eight life areas would bring change, since that were not part of my routine clinical practice so far. I think that structured conversation would allow us to discuss more things in less time and to be sure that we didn’t miss any important life area.

### Frequency of intervention delivery

Some participants provided brief answers about the frequency of DIALOG+ delivery. Once per month was the most common view, particularly among clinicians and policymakers. Some patients and carers requested more frequent delivery, for instance weekly or bi-weekly. Less frequent delivery, such as “once in two months” and “at least twice a year”, was favoured by some other patients and carers. From participants’ accounts it was clear that patients preferred more frequent use compared to clinicians and policymakers. Carer38 proposed DIALOG+ be delivered every 6 months because changes to patients’ life satisfaction take time. Carer37 expressed that DIALOG+ “should be applied regularly during outpatient appointments”. These accounts suggest that the frequency of DIALOG+ sessions should be determined by the individual patient’s needs.

### Suggested changes to the intervention

Some participants’ accounts contained broad recommendations about changes to DIALOG+, however these were not explored in detail. Suggestions about the DIALOG scale were made, such as adding a miscellaneous field so patients can discuss topics not included in the scale, and reducing it to a 5-point Likert scale. Clinician46 expressed that “it might be practical and easy to use smartphones for [delivering DIALOG+]”. About the area ‘job situation’, policymaker4 said “it should be clearer that it also refers to education, or how the patient is dealing with being unemployed.” Clinician36 spoke about the longer time needed to see changes in patients’ satisfaction with areas such as ‘job situation’ and ‘accommodation’ than that with their mental health, thus “perhaps these should be assessed only at a 6-month interval.”

## Discussion

Our study’s findings offer important implications for implementation of digital psychosocial intervention for PSD in low-resource settings.

Our results show that resources regarding time, staff, funding and space for the implementation of a novel intervention in the explored services are perceived as limited. Lack of financial and human resources have been found to be key implementation roadblocks for digital interventions for people with psychosis [19]. The cost of tablets overall was not considered to be a major burden in the implementation of DIALOG+ in the context of SEE. Rather, participants’ accounts focused more on the lack of human resources. Special consideration should be provided to psychiatrists’ time limitations that arise as a result of limited human resources for the number of patients, because this barrier was mentioned across stakeholders and countries. To maximize its sustainability, DIALOG+ should not require much longer clinical meetings with psychiatrists and other mental health professionals than usual. The findings suggest inclusion of other mental health professionals in the implementation process of DIALOG+ to address the limited availability of psychiatrists in most of the participating countries. Such suggestions were also given by the LEAP and support the stepped care approach in mental health services [26], especially in contexts with limited human resources. Digital support tools have been reported to facilitate a diverse set of mental health workers in delivering the care needed by patients with severe mental illnesses in the community, which is of particular necessity in LMICs [27].

Attractive intervention characteristics, such as clarity and easiness of use, and ability to provide structure to patient-clinician meetings, involve patient’s psychosocial needs and track patient’s progress were implementation facilitators considered important by the key stakeholders. ‘Perceptions of the innovation’ has been identified as one of the basic determinants associated with the rate of implementation of the intervention [28], thus it is paramount that key stakeholders have positive perceptions of the intervention. The findings also support previous reports that the comprehensive structure enabled by DIALOG+ is one of its mechanisms of action [10]. Automated data collection offered by digital psychosocial interventions is useful to systematically measure the quality of psychosocial care, a shortcoming identified with current psychosocial therapies [27].

Despite the identified implementation facilitators, the findings show the need to consider the patients’ level of existent familiarity with life and treatment satisfaction questionnaires using Likert scales when introducing the intervention, as a few patients found the DIALOG scale unclear. Patients who used DIALOG+ in the UK also reported ‘difficulty to understand or cope with questions’, although this was not explored in detail [10]. Similarly, a systematic review of implementation determinants for digital interventions for people with psychosis recognized the complexity of the intervention as an impediment for those with psychiatric symptoms, low intelligence quotient and low technological competence [19]. Some clinicians and patients directly expressed a need to receive more training, largely explained by receiving only a short presentation of the intervention prior to the focus group discussions and some patient’s lack of familiarity with smart phones. Thus, besides clinicians, patients may also need training to use the intervention as planned. Such training could be delivered by the clinicians who can explain key principles and procedures to the patient during their first sessions.

Additionally, many positive attitudes and sufficient level of preparedness for DIALOG+ implementation across potential intervention adopters was reported, particularly related to the perceived existence of the required skills and high stakeholders’ motivation. Such implementation facilitators are of significance because negative attitudes and scepticism have been shown to reduce motivation to adopt the interventions for both clinicians and patients with psychosis [19].

Some participants raised concerns about potential resistance to innovation in mental health care by clinicians, particularly to the technological aspect of the intervention as use of digital interventions is not part of routine mental health care in the SEE countries that participated in this study. A study exploring the experiences of using DIALOG+ in the UK also identified ‘initial apprehension to the technology’ as a barrier [10]. The LEAP similarly stressed that clinicians need to be adequately trained, motivated and willing to use DIALOG+, while not having any resistance. Thus, the potential resistance to innovation should be considered when developing training for digital interventions. Moreover, carers and policymakers expressed concerns that poor mental health would limit patients’ engagement and thus negatively impact DIALOG+ implementation, further highlighting that the intervention may not be suitable for all patients and clinicians. Accessibility and adaptability of digital interventions for people with psychosis and treating clinicians are reported as main implementation facilitators of digital interventions identified in previous studies [19] and need to be carefully considered.

Despite the views of potential resistance to innovation, key stakeholders perceived the intervention as needed to improve the scope of the conversations during routine clinical meetings. This is promising because stakeholders not perceiving that a change is needed is an important barrier to innovation implementation in mental health care [28].

DIALOG+ was mostly seen as consistent with existing norms and practices, an aspect that is often reported among strong enablers of innovation implementation across health care [29, 30]. However, some important opposing views regarding the incompatibility of DIALOG+ with the mental healthcare funding policy in North Macedonia and Serbia and the lack of intervention flexibility should be considered. Similarly, the LEAP highlighted that asking questions in a mechanical way could make the sessions ‘boring and overwhelming’. Previous research in the UK similarly identified repetitiveness of DIALOG+ as a barrier [10]. This result highlights the importance of considering individual patient’s needs regarding how frequently the intervention is used to avoid repetitiveness. The UK trial of DIALOG+ suggested that the intervention be used monthly for 3 months and flexibly afterwards [8]. However, the initial perception among participants in this study was that the intervention should be delivered once a month.

Clinicians expressed that institutional support is a strong determinant for successful DIALOG+ implementation. This insight adds to the limited evidence of contextual and organisational factors regarding implementation of digital interventions for PSD [19].

Different opinions on the way and the extent to which carers should be involved were expressed. The findings together with the LEAP feedback indicate that it is important to involve carers in the implementation process and develop their specific role through discussion between clinicians, patients and carers.

Increased patient empowerment and strengthened clinician-patient relationship were considered to be important potential individual benefits of DIALOG+ by all key stakeholders. This is in line with results from the UK study researching mechanisms of action of DIALOG+ [10]. The opportunity of DIALOG+ to shift the typical medical approach to a more psychosocial approach – an approach that was recognized by all stakeholders as often neglected – was expressed as a potential organisational benefit. In this approach, patients would be at the centre of their treatment and DIALOG+ would offer improved structure and follow up of sessions. These views were also shared by the LEAP. The identified potential benefits from participants’ accounts present important facilitators to further the implementation process of digital psychosocial interventions in the explored context in SEE.

Digital interventions are becoming an integral part of the transformation of mental health care because of their potential to advance current services and to enable new pathways for provision of innovative psychosocial therapies [31, 32]. The major implementation barrier identified in our current study was psychiatrists’ time constrains, which is consistent with other LMIC settings considering implementation of digital psychosocial interventions. The process of implementing a digital intervention can be time consuming as it requires changes to routine services at an individual and an organisational level that frequently do not occur successively [33]. The collaboration between key stakeholders in the development and implementation of digital interventions is a crucial promoter of implementation of digital interventions for people with psychosis [19]. In order for technology to meaningfully ‘disrupt’ the current approach to mental health care, it needs to be inclusive of people with severe mental illnesses, consider how mental health providers can use such technologies most adequately and develop digital interventions with having low-resource settings in mind.

### Strengths and limitations

A major strength of this study was the inclusion of perspectives from four key stakeholder groups and feedback from LEAP across five LMICs in SEE, which is rarely seen in the literature. Early communication with key stakeholders is crucial to innovation development and successful implementation as it enables an increased understating of the context in which intervention implementation occurs [34]. The findings from this study contribute to the field of implementation science because they provide insight into currently limited knowledge of perceived facilitators and barriers of implementing a digital psychosocial health intervention in low-resource settings for PSD.

The study has several limitations. Participants’ responses about changes to DIALOG+ and its delivery frequency were not explored in detail during the focus groups, limiting the subsequent data analysis. Nonetheless, these data were included since this they were related to the study’s research aim; however future research should explore the related themes further. Although all focus groups facilitators received training and had topic guides, the quality and depth of information varied across focus groups. This might have occurred because of various styles of individual facilitators and differences in their background (e.g., medical vs. psychological), as well as because of the varied motivation of participants to contribute to a theme. This is often reported as a challenge when conducting cross-national and cross-disciplinary qualitative research as each team member has their own combination of theoretical, national and linguistic underpinnings, as well as varying levels of methodological experience [35]. Additionally, all transcripts were translated into English from five different languages, which introduced a risk of losing contextual-specific meaning and traits [35]. However, this was a pragmatic decision as early stage translation enabled collaboration in the analysis process within the research team. The transcripts were not back-translated or checked with focus group participants; thereby, the process of translating transcripts could have introduced a bias that might have influenced the analysis of transcripts. However, the analysis team was in regular contact with focus group facilitators to clarify any ambiguous meaning of words. The analysis team consisted of researchers that were both proficient in English and some of the SEE languages and were able to go back to original transcripts where necessary. Additional limitation of the study was not analysing data after each focus group in order to determine the saturation of data. To future cross-national, multi-disciplinary research teams conducting qualitative studies, we recommend in-depth training in qualitative methods, involving researchers from all countries in the analysis process and maintaining transcripts in their original languages. We hope that our approach and lessons learned can contribute to the literature on conducting such qualitative research.

## Conclusion

This study provides increased understanding from different stakeholders’ perspectives regarding the implementation of a digital psychosocial intervention for PSD in five LMICs in SEE. Implementation of DIALOG+ is supported by the key stakeholders, who overwhelmingly reported implementation facilitators compared to barriers, and as such is promising to advance care for PSD patients in SEE. DIALOG+ fits well with the organisational goal of the mental health centres of interest, but would require transformation of the nature of routine clinical meetings in mental health care, which can be challenging for both clinicians and patients. Identification of potential barriers and facilitators promotes successful uptake of the intervention, minimizes waste of scarce resources and provides evidence about the provision of digital psychosocial treatment for PSD in LMICs. Working towards implementation of DIALOG+ is an opportunity to move beyond lip service that mental health services need to pay more attentions to psychosocial aspects. These findings, together with the existing evidence base, provide support for further examination of the effectiveness and implementation of DIALOG+ in LMICs in SEE with the expected impact of improving management of PSD in this region.

## Supplementary Information

**Additional file 1 **DIALOG+ App. **Figure 1**. A visual representation of how DIALOG+ app displays the 11 life domain ratings, allowing comparison of ratings with those from previous sessions. The ticks on the right mark the domains selected for further discussion in the four-step solution-focused approach (DIALOG+ Manual, https://dialog.elft.nhs.uk/East London, accessed 7 July 2020). **Figure 2.** A visual representation of the DIALOG+ screen showing the four-step solution-focused approach (DIALOG+ Manual, https://dialog.elft.nhs.uk/East London, accessed 7 July 2020).

**Additional file 2.** Reporting checklist for qualitative study. Based on the SRQR guidelines.

**Additional file 3.** Topic Guides.

**Additional file 4.** Codes used for coding.

## Data Availability

The dataset (which includes individual transcripts) used and analysed during the current study is not publicly available due to confidentiality policies. In case someone request the data from this study they should contact the Principal Investigator and Nikolina Jovanović at n.jovanovic@qmul.ac.uk

## Abbreviations

PSD: Psychosis spectrum disorders
SEE: Southeast Europe
LMICs: Low- and middle-income countries
LEAP: Lived Experience Advisory Panel

## Footnotes

1 Kosovo is referred throughout the text by United Nations resolution.
