# Supplementary material for: Implementing a digital mental health intervention for individuals with psychosis - a multi-country qualitative study
Source: BMC Psychiatry. 2021 Sep 25;21:468. doi: 10.1186/s12888-021-03466-x (PMC8466399; doi:10.1186/s12888-021-03466-x)
Supplement: Supplementary file 1 — Additional file 1 DIALOG+ App. Figure 1. A visual representation of how DIALOG+ app displays the 11 life domain ratings, allowing comparison of ratings with those from previous sessions. The ticks on the right mark the domains selected for further discussion in the four-step solution-focused approach (DIALOG+ Manual, https://dialog.elft.nhs.uk/East London, accessed 7 July 2020). Figure 2. A visual representation of the DIALOG+ screen showing the four-step solution-focused approach (DIALOG+ Manual, https://dialog.elft.nhs.uk/East London, accessed 7 July 2020). [file 12888_2021_3466_MOESM1_ESM.docx]

# Additional File 1

# DIALOG+ App

**Figure 1.** A visual representation of how DIALOG+ app displays the 11 life domain ratings, allowing comparison of ratings with those from previous sessions. The ticks on the right mark the domains selected for further discussion in the four-step solution-focused approach (DIALOG+ Manual, https://dialog.elft.nhs.uk/East London, accessed 7 July 2020).

**Figure 2.** A visual representation of the DIALOG+ screen showing the four-step solution-focused approach (DIALOG+ Manual, https://dialog.elft.nhs.uk/East London, accessed 7 July 2020).
