# Supplementary material for: Implementing a digital mental health intervention for individuals with psychosis - a multi-country qualitative study
Source: BMC Psychiatry. 2021 Sep 25;21:468. doi: 10.1186/s12888-021-03466-x (PMC8466399; doi:10.1186/s12888-021-03466-x)
Supplement: Supplementary file 3 — Additional file 3. Topic Guides. [file 12888_2021_3466_MOESM3_ESM.docx]

**Additional File 3**

**Topic Guides**

| \| \| \| **INTERVIEW/FOCUS GROUP GUIDE: Patients and family members** \| \| --- \| \| \| --- \| --- \|     **Facilitator’s welcome, introduction and instructions to participants**  ***Welcome*** *and thank you for participating in this focus group/interview. You have been asked to participate as your point of view is important. We really appreciate your time. Introduce anyone else from team if present in the room.*  **Introduction:** Explain that focus group discussion is designed to assess your current thoughts and feelings about the Dialog + intervention that has been designed to make routine meetings between clinicians and patients structured, comprehensive and more therapeutically effective.  *We will show you DIALOG+ in a little while. The meeting can last up to 90 minutes. We would like to tape the discussion to facilitate recollection* (switch on the recorder).  **Anonymity:** Ensure the participants on the anonymity of the discussion. Ask them to try to answer and comment as accurately and truthfully as possible. When talking about staff members or other patients, you do not have to use their names.  **Ground rules**   - *The most important rule is that only one person speaks at a time. There may be a temptation to jump in when someone is talking but please wait until they have finished.* - *There are no right or wrong answers* - *You do not have to speak in any particular order* - *When you do have something to say, please do so. There are many of you in the group and it is important that I obtain the views of each of you* - *You do not have to agree with the views of other people in the group* - *Does anyone have any questions?* - *OK, let’s begin*   **Warm up**   - *First, I’d like everyone to introduce themselves. Can you tell us your name?*   **Introductory question**  *I am just going to give a short presentation of what is Dialog+ so we should all be in common ground and continue with discussion.*  **Guiding questions:**  1. WHAT ARE YOUR INITIAL THOUGHTS OF DIALOG+ (Knowledge)   - *What did you like/dislike?* - *Are the life domains clear and easy to understand?* - *Was the rating scale easy to use?*   2. THINKING OF THE SERVICES THAT YOU (OR YOUR FAMILY MEMBER) RECEIVE, HOW IS THIS DIFFERENT OR THE SAME? (Knowledge)   - *Do you think the conversation or areas covered in your appointments would be different? How? Why?* - *If yes, how do you feel about the changes?* - *If no, are there things that you would like to have changed?* - *Do you think you would talk to your clinicians differently? In what way* - *Can you see DIALOG + being implemented regularly/consistently in clinical setting that you visit (your family member visits)?*   3. WHAT ARE THE POTENTIAL BENEFITS OF IMPLEMENTING DIALOG+ FOR YOU AS A PATIENT? (Beliefs about consequences)   - *How do you think it would be useful to patients?* - *How often you would like to use it?* - *Why do you think X would be a benefit?* - *Would you feel comfortable in receiving (family member receiving) DIALOG+?*   4. WHAT ARE THE POTENTIAL DIFFICULTIES OR BARRIERS OF IMPLEMENTING DIALOG+? (Beliefs about consequences)   - *Is the time allowed for routine appointments sufficient?* - *Are there resources in place to implement DIALOG+?* - *Why do you think X would be difficult?* - *How could we overcome these barriers?*   5. DO YOU THINK THAT CLINICIANS / STAFF WOULD BE EAGER AND OPEN TO BE TRAINED IN DIALOG+ (Social influences / Beliefs about consequences)   - *Do you think it would be easy or difficult to make DIALOG+ a regular part of your meetings with clinicians in mental health services?* - *If difficult, please tell us more about the reasons* - *What would other patients (family members would) think of DIALOG+?*   6. WHAT SKILLS ARE NEEDED TO USE DIALOG+? (Skills)   - *What skills and/or information would you need?* - *What skills would clinicians need?* - *Do you have any similar experiences with interventions such as DIALOG+ (received by your family member)?*   7. WHAT DO YOU THINK ABOUT THE ROLE OF CARERS IN DIALOG+ INTERVENTION?   - *What role do you feel carers could or should play in DIALOG+?* - *Do you think it would be easy or difficult to engage carers in helping patients to use DIALOG+ why?* - *Do you think it would be easy or difficult for patients to complete the agreed tasks & activities between meetings? Why?*   8. WHAT WOULD MAKE IT EASIER TO USE DIALOG+ (FOR YOUR FAMILY MEMBER TO USE DIALOG+) (Behavioral regulation)   - *How would that make it easier?* - *What would make it easier for services and clinicians to use it?*   **Concluding question**   - *Of all the things we’ve discussed today, what would you say are the most important issues you would like to express about DIALOG+?* [Go round the group and ask everyone to say one key improvement or one final thought]   **Conclusion**   - *Thank you for participating. This has been a very successful discussion* - *Your opinions will be a valuable asset to the study* - *We hope you have found the discussion interesting*   Please use probes during the process  **General probes to be used during the focus group**  “Tell me more about that.”  “What is it like?”  “What led you to… (feel, think in specific way). . . “  “Compare this to what others have done.”  “Can you think of another example of this?”  “Give me more details please.”  “I’d like to hear more.”  “How did that make you feel?” \|  \|  \| \| --- \| --- \| --- \| --- \| --- \| |
| --- | --- | --- | --- | --- | --- |

|  |  |  |
| --- | --- | --- |
|  | |  |
|  | |  |

| \| \| \| **INTERVIEW/FOCUS GROUP GUIDE: Clinicians and services providers** \| \| --- \| \| \| --- \| --- \|     **Facilitator’s welcome, introduction and instructions to participants**  ***Welcome*** *and thank you for participating in this focus group/interview. You have been asked to participate as your point of view is important. We realise you are busy and we really appreciate your time.*  Introduce anyone else from team if present in the room.  **Introduction:** Explain that focus group discussion is designed to assess your current thoughts and feelings about the DIALOG+ intervention that has been designed to make routine meetings between clinicians and patients structured, comprehensive and more therapeutically effective.  *We will present DIALOG+ to you in a short while. The meeting can last up to 90 minutes. We would like to tape the discussion to facilitate recollection* (switch on the recorder).  **Anonymity:** Ensure the participants on the anonymity of the discussion. Ask them to try to answer and comment as accurately and truthfully as possible. When talking about staff members or other patients, you do not have to use their names.  **Ground rules**   - *The most important rule is that only one person speaks at a time. There may be a temptation to jump in when someone is talking but please wait until they have finished.* - *There are no right or wrong answers* - *You do not have to speak in any particular order* - *When you do have something to say, please do so. There are many of you in the group and it is important that I obtain the views of each of you* - *You do not have to agree with the views of other people in the group* - *Does anyone have any questions?* - *OK, let’s begin*   **Warm up**   - *First, I’d like everyone to introduce themselves. Can you tell us your name?*   **Introductory question**  *I am just going to give a short presentation of what is DIALOG+ so we should all be in common ground and continue with discussion*  **Guiding questions**  1. WHAT ARE YOUR INITIAL THOUGHTS ON THE DIALOG+ INTERVENTION? (Knowledge)   - *What did you like/dislike?* - *Are the life domains clear and easy to understand?* - *Was the rating scale easy to use*? - *Did the four-step approach makes sense?*   2. THINKING OF THE SERVICES YOU PROVIDE, HOW IS THIS DIFFERENT OR THE SAME? (Nature of the behaviour)   - *Do you think the conversation or areas covered in your usual appointments would be different from the areas covered by DIALOG+? How? Why?* - *If yes, how do you feel about the changes?* - *If no, are there things that you would like to have changed?* - *Do you think you would talk to your patients differently? In what way* - *Can you see DIALOG + being implemented regularly/consistently in your clinical setting?* (Behavioural regulation)   3. THINKING OF ALL THE WORK AND PRIORITIES YOU HAVE, HOW IMPORTANT DO YOU THINK IT IS TO DELIVER DIALOG+? (Motivation and goals?)   - *Would you consider the DIALOG +to be part of your clinical responsibility?* (Social/Professional role and identity) - *What would your colleagues think of delivering DIALOG+* (Social influences) - *What would your patients think about DIALOG+?* (Social influences)   4. WHAT ARE THE POTENTIAL BENEFITS OF IMPLEMENTING DIALOG+ (Beliefs about consequences)   - *How do you think it would be useful to patients?* - *Are there any service level or benefits for the clinicians?* - *Why do you think X would be a benefit?*   5. WHAT ARE THE POTENTIAL DIFFICULTIES OR BARRIERS OF IMPLEMENTING DIALOG+?   - *How does it fit with existing policies and treatment protocols?* (Nature of the behaviour) - *Is the time allowed for routine appointments sufficient?* (Environmental context and resources) - *Are there resources in place to implement DIALOG+?* (Environmental context & resources) - *Why do you think X would be difficult?* (Beliefs about capabilities) - *What type of support or strategies would be helpful?* (Behavioural regulation)   6. DO YOU THINK THAT CLINICIANS WOULD BE EAGER AND OPEN TO BE TRAINED IN DIALOG+ (Social influences / beliefs about consequences)   - *How would DIALOG+ fit with your existing training and continued professional development? (*Social/Professional role and identity) - *Do you think it would be easy or difficult to make DIALOG+ a regular part of your meetings with patients in mental health services? Why?* (Beliefs about capabilities) - *Would you feel comfortable in delivering DIALOG+?* (Skills) - *How could your existing team meetings and supervision support implementation* (Environmental context and resources)   7. WHAT SKILLS ARE NEEDED TO USE DIALOG+? (Skills)   - *What skills and training would you need to implement it?* (Skills) - *Do you have any similar experiences as Dialog +?* (Social influences) - *Is DIALOG+ manual easy to understand?* (for pilot clinicians only) - *Do you think that the training (online and face-to-face) is acceptable?* (for pilot clinicians only) - *Was the training sufficient? If not why not?*   8. WHAT DO YOU THINK ABOUT THE ROLE OF CARERS IN DIALOG+ INTERVENTION? (Social influences)   - *What role do you feel carers could or should play in DIALOG+?* - *Do you think it would be easy or difficult to engage carers in helping patients to use DIALOG+ why?* - *Do you think it would be easy or difficult for patients to complete the agreed tasks & activities between meetings? Why?*   9. WHAT WOULD MAKE IT EASIER TO USE DIALOG+ IN YOUR CURRENT PRACTICE (Behavioral regulation)   - *How would that make it easier?* - *What else would you like to know about DIALOG+?* (Knowledge)   **Concluding question**   - *Of all the things we’ve discussed today, what would you say are the most important issues you would like to express about DIALOG+?* [Go round the group and ask everyone to say one key improvement or one final thought]   **Conclusion**   - *Thank you for participating. This has been a very successful discussion* - *Your opinions will be a valuable asset to the study* - *We hope you have found the discussion interesting*   Please use probes during the process  **Probes**  “Tell me more about that.”  “What is it like?”  “What led you to (feel, think in specific way). . . “  “Compare this to what others have done.”  “Can you think of another example of this?”  “Give me more details please.”  “I’d like to hear more.”  “How did that make you feel?” \|  \|  \| \| --- \| --- \| --- \| --- \| --- \| |
| --- | --- | --- | --- | --- | --- |
