# Supplementary material for: Implementing a digital mental health intervention for individuals with psychosis - a multi-country qualitative study
Source: BMC Psychiatry. 2021 Sep 25;21:468. doi: 10.1186/s12888-021-03466-x (PMC8466399; doi:10.1186/s12888-021-03466-x)
Supplement: Supplementary file 4 — Additional file 4. Codes used for coding. [file 12888_2021_3466_MOESM4_ESM.docx]

**Additional file 4 – codes used for coding**

**Perceived benefits of D+**

1. **Perceived benefits of D+**
2. **Improved mental health care practice**
3. **Improved clinician-patient relationship/communication**
4. **Patient empowerment**
5. **Improved patient outcomes**
6. **Patient involvement in treatment**
7. **Ability to track patient state and progress**
8. **Perceived need for D+**

**Perceived barriers to D+**

1. **Perceived barriers to D+**
2. **Limited time**
3. **Limited funding**
4. **Limited human resources**
5. **No tablets**
6. **Conflicting with priorities/norms/policies**
7. **Prejudice/stigma/marginalization of psychosis**
8. **Patient trust issues**
9. **Limited physical space**

**Perceived facilitators of D+**

1. **Perceived facilitators of D+**
2. **Clinician skills related to D+**
3. **Fits with existing practice & professional role**
4. **Caregivers’ involvement in D+**
5. **Consistent with priorities/norms/policies**
6. **Study effects of D+**
7. **Acceptability of solution focused approach**
8. **Patient mental state and usage of D+**
9. **Clarity of scales**
10. **Willingness to use/uptake D+**
11. **Clarity of focus on solutions clarity of domains**
12. **Usability of D+**

**Others**

1. **Suggested changes to D+**
2. **Frequency of D+ use**
3. **Ethical considerations**
4. **Professional roles/responsibilities/suggestions who to use**
5. **Caregiver involvement in D+**
6. **Patient emotional needs**
